# Catheter-Associated Soft Tissue Infection Caused by *Pantoea piersonii* in a Totally Implantable Venous Access Port: The First Report from China

**DOI:** 10.3390/microorganisms14091996

**Published:** 2026-09-09

**Authors:** Yachun Zhou, Anmin Ren, Xianting Huang, Ashao Jia, Yingfeng Huang, Pingping Chen, Ruoyu Jia, Li Deng, Liang Yang, Xianyuan Zhou, Weidong Zheng

**Affiliations:** 1 Department of Laboratory Medicine, Shenzhen University General Hospital, Shenzhen 518000, China; 2 Joint Laboratory of Guangdong-Hong Kong Universities for Vascular Homeostasis and Diseases, Department of Pharmacology, School of Medicine, Southern University of Science and Technology, Shenzhen 518055, China

**Keywords:** *Pantoea piersonii*, implantable port, soft tissue infection, MALDI-TOF, genome sequencing

## Abstract

*Pantoea piersonii* (formerly *Kalamiella piersonii*) is a recently reclassified environmental Gram-negative bacterium belonging to the family Erwiniaceae. Human infections caused by this organism are extremely rare. Here, we report a rare case of implantable venous access port-associated soft tissue infection caused by *P. piersonii* in a 49-year-old immunocompromised woman with lung cancer. The pathogen was isolated from purulent discharge obtained from the port site and identified by matrix-assisted laser desorption/ionization time-of-flight mass spectrometry (MALDI-TOF MS). Whole-genome sequencing confirmed species-level identification based on average nucleotide identity (ANI) analysis and phylogenomic reconstruction. The isolate exhibited broad antimicrobial susceptibility to the tested agents. Following complete device removal and empirical antimicrobial therapy, the patient recovered uneventfully. This study reports the first documented case of *P. piersonii* infection in China and provides a comprehensive clinical and genomic characterization of a newly isolated clinical strain.

## 1. Introduction

The genus *Pantoea* comprises a diverse group of Gram-negative bacteria widely distributed in environmental, plant-associated, and opportunistic clinical settings [1,2]. Although many species within this genus are considered environmental organisms, increasing evidence suggests that certain members can act as opportunistic pathogens, particularly in immunocompromised patients or those with indwelling medical devices [3,4]. *Pantoea piersonii* (*P. piersonii*) is a relatively recently described species within the genus [5,6]. It was first isolated from the International Space Station in 2019 and initially classified as *Kalamiella piersonii (K. piersonii)*. Subsequent phylogenomic analyses supported its reclassification into the genus *Pantoea* [7]. Human infections caused by this organism are exceedingly rare, with only a few cases reported to date involving bloodstream, urinary tract, gastrointestinal, and cardiovascular infections [5,6].

Here, we report a rare case of catheter-associated infection caused by *P. piersonii* in a patient with advanced malignancy. The isolate was identified using matrix-assisted laser desorption/ionization time-of-flight mass spectrometry (MALDI-TOF MS) and further characterized by whole-genome sequencing, including average nucleotide identity (ANI) analysis, phylogenomic reconstruction, synteny comparison, and resistome profiling. This study aims to expand the current understanding of the clinical relevance and genomic features of *P. piersonii* and provides the first documented case of this organism in China.

## 2. Case Description

A 49-year-old woman was admitted on 24 April 2025 due to catheter-related thrombosis detected in the right upper limb. The patient had received implantation of a right upper limb venous access port in November 2021. Her medical history included stage IV lung adenocarcinoma with bone metastasis. She had previously received multiple courses of systemic anticancer therapy, including pemetrexed plus carboplatin in combination with bevacizumab and zoledronic acid, during which she developed grade IV chemotherapy-induced myelosuppression requiring subsequent dose reduction of carboplatin. She also underwent local radiotherapy for an intracranial lesion in February 2023, followed by maintenance anticancer therapy with pemetrexed plus bevacizumab and subsequently bevacizumab plus nab-paclitaxel. On admission, the patient was afebrile without signs of local infection. Laboratory tests revealed a white blood cell count of 4.61 × 10^9^/L (neutrophils 78.3%), C-reactive protein (CRP) of 10.32 mg/L, and procalcitonin of 0.192 ng/mL. Ultrasound confirmed thrombosis of the right upper extremity veins. Preoperative assessment indicated high risk for venous thromboembolism (score: 8) and poor nutritional status. The patient’s overall health was suboptimal. However, a standardized performance status was not documented during this hospitalization. In addition, the patient was not receiving cytotoxic anticancer chemotherapy at the time of the present admission in April 2025.

The catheter tip was located at the junction between the lower third of the superior vena cava and the right atrium. The port reservoir was implanted in a subcutaneous pocket of the right upper arm along the basilic vein, with a catheter length of 40 cm. During surgical removal of the port on 29 April 2025, erythema was observed around the port site. A 0.5 cm skin defect with approximately 1 mL of gray-white purulent discharge was identified and collected for culture. Complete device removal was achieved under fluoroscopic guidance due to severe adhesion. Intraoperative blood loss was approximately 5 mL. Postoperatively, empirical intravenous antimicrobial therapy with piperacillin-tazobactam (4.5 g every 12 h) was initiated on 29 April 2025. Piperacillin-tazobactam was discontinued on April 30, and oral cefuroxime axetil (0.25 g every 12 h) was initiated and continued for two weeks. Complete removal of the implantable venous access port may provide definitive source control. The patient remained clinically stable, with uneventful wound healing and no subsequent complications.

## 3. Materials and Methods

### 3.1. Antimicrobial Susceptibility Testing

Antimicrobial susceptibility testing was performed using the BD Phoenix 50 automated microbiology system (Becton, Dickinson and Company [BD], Franklin Lakes, NJ, USA) with the NMIC-413 Gram-negative susceptibility panel. A pure bacterial colony was suspended to a turbidity equivalent to a 0.5 McFarland standard and tested according to the manufacturer’s instructions using software version V2.80.0.0. Because *Pantoea* belongs to the order Enterobacterales, Minimum inhibitory concentration (MIC) results for most antimicrobial agents were interpreted according to CLSI M100, 34th edition, breakpoints for Enterobacterales. Tigecycline was interpreted according to U.S. Food and Drug Administration (FDA) breakpoints, whereas colistin was interpreted according to EUCAST version 10.0 criteria. For cefoperazone/sulbactam, for which no specific CLSI interpretive breakpoint is available, the result was interpreted in our laboratory with reference to the cefoperazone breakpoint.

### 3.2. MALDI-TOF MS Analysis

Briefly, a well-isolated colony was evenly smeared onto a designated spot of the MALDI target plate. The sample was overlaid with 1 μL of 70% formic acid and allowed to air-dry. Subsequently, 1 μL of matrix solution was applied to the same spot and allowed to dry completely at room temperature. The prepared target plate was then introduced into the MALDI-TOF (Bruker Microflex LT/SH, Bremen, Germany) mass spectrometer for analysis. The resulting mass spectrum was analyzed using the instrument-associated identification system for species identification.

### 3.3. Whole-Genome Sequencing, Assembly, and Quality Assessment

Whole-genome sequencing was performed using a combination of PacBio long-read and Illumina short-read sequencing. PacBio HiFi sequencing was performed on a Revio platform (SMRT Link v13.0; CCS v8.2.0), yielding 17,334 HiFi reads (read N50 15,592 bp; ~49× genome coverage). Illumina sequencing was performed on a NovaSeq X platform (150-bp paired-end, 350-bp insert), yielding 5,397,602 read pairs (~1.62 Gb raw; 1.35 Gb clean, Q30 96.77%). Assembly statistics were calculated with QUAST v5.3.0; genome completeness and contamination were assessed with CheckM2 v1.1.0, and completeness was further supported by BUSCO (99.2% of 124 single-copy orthologs). Replicon circularity was verified by mapping both Illumina and PacBio HiFi reads to the assembly with minimap2 v2.28, and counting reads spanning the sequence-end junction of each replicon: the chromosome and plasmids pSZUGH1-1, -3, and -4 were supported as circular by both data types, whereas pSZUGH1-2 could not be circularized.

### 3.4. Genome Annotation and Resistance/Virulence Gene Prediction

Genome annotation was performed using Bakta v1.12.1 with database v6.0 (light). Putative virulence factors and antimicrobial resistance genes were identified by screening the annotated genome against the VFDB and CARD databases using ABRicate v1.4.0.

### 3.5. Comparative Genomic and Phylogenetic Analyses

A total of 127 publicly available *Pantoea* and related genomes were downloaded from NCBI (accession numbers in Supplementary Table S1). Pairwise ANI values were calculated using fastANI v1.34 (k-mer size 16, fragment length 3000 bp). For synteny analysis, each reference genome was aligned to the SZUGH1 chromosome using NUCmer v4.0 (MUMmer package; delta-filter -1 -l 1000), and the results were visualized with pyCirclize v1.10.1. For phylogenetic reconstruction, orthologous gene clusters were inferred with Panaroo v1.8.0, core genes were aligned with MAFFT v7.526, and a maximum-likelihood tree was reconstructed with IQ-TREE v3.1.3 (-m MFP; best-fit model GTR+F+I+R7 selected by BIC; 1000 ultrafast bootstrap replicates) from the concatenated alignment of 1051 core genes (present in ≥99% of genomes and passing alignment quality filtering), with Duffyella gerundensis EM595 as the outgroup. Isolation-source metadata were retrieved from the corresponding BioSample records.

## 4. Isolate Characterization

**Figure 1:**
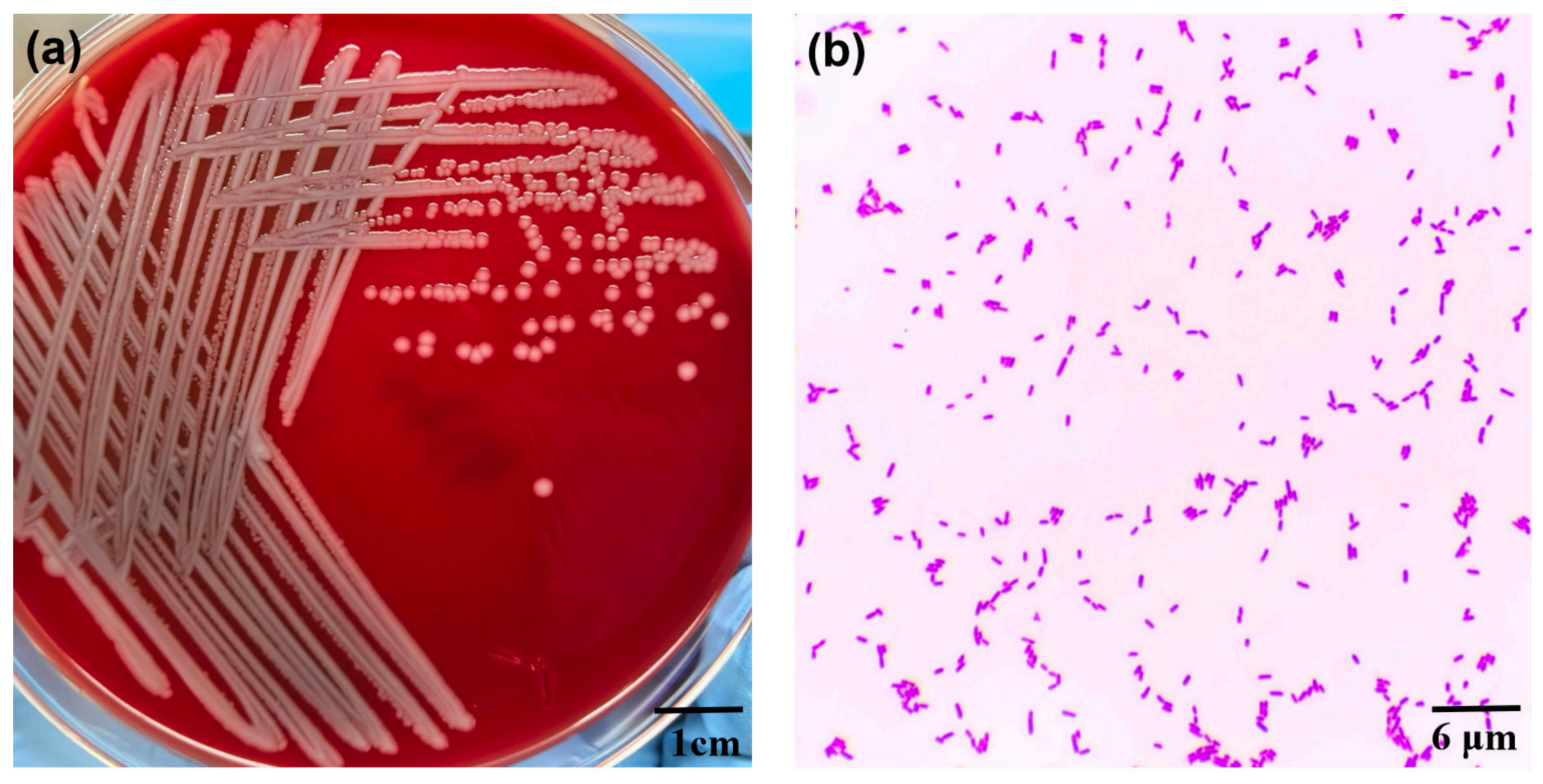
Morphological characteristics of *P. piersonii*. (**a**) Colony morphology of *P. piersonii* grown on blood agar, showing smooth, circular, convex colonies with a creamy-white appearance after incubation, scale bar = 1 cm. (**b**) Gram-stained microscopic image of *P. piersonii* revealing Gram-negative pink-stained cells predominantly arranged in small clusters and short chains under oil microscopy, scale bar = 6 µm.

**Figure 2:**
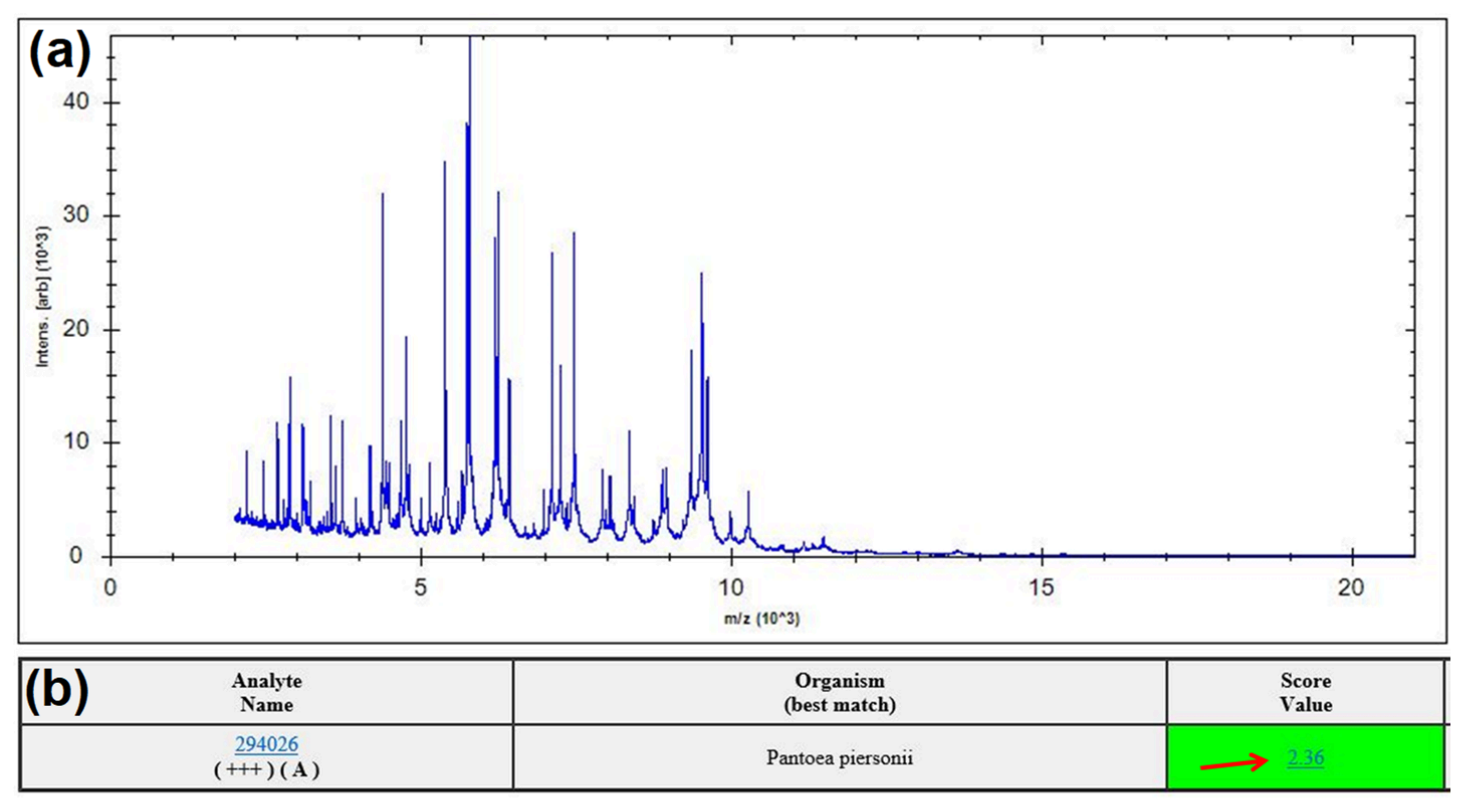
MALDI-TOF MS identification of *P. piersonii SZUGH1*. (**a**) Representative MALDI-TOF mass spectrum of the clinical isolate. (**b**) MALDI-TOF MS identification score graph showing species-level identification of the isolate as *P. piersonii*.

Purulent specimens were aseptically collected from the periprosthetic area of the implantable venous access port during surgery on 29 April 2025. Samples were immediately inoculated onto Columbia blood agar and incubated at 35 °C in a 5% CO_2_-enriched aerobic atmosphere. After 24 h of incubation, visible bacterial growth was observed (30 April 2025). On Columbia blood agar, colonies appeared as abundant, smooth, convex, opaque white colonies with regular margins and a moist surface, without evidence of β-hemolysis (Figure 1a). Gram staining demonstrated medium-sized Gram-negative short rods (Figure 1b). A single well-isolated colony was selected for species identification using MALDI-TOF MS (Figure 2a). The isolate was identified as *P. piersonii* with a high-confidence score of 2.36 (Figure 2b). As this represents the first reported case of *P. piersonii* infection in China, the isolate was designated *P. piersonii* Shenzhen University General Hospital 1 (*P. piersonii* SZUGH1) throughout this study.

**Figure 3:**
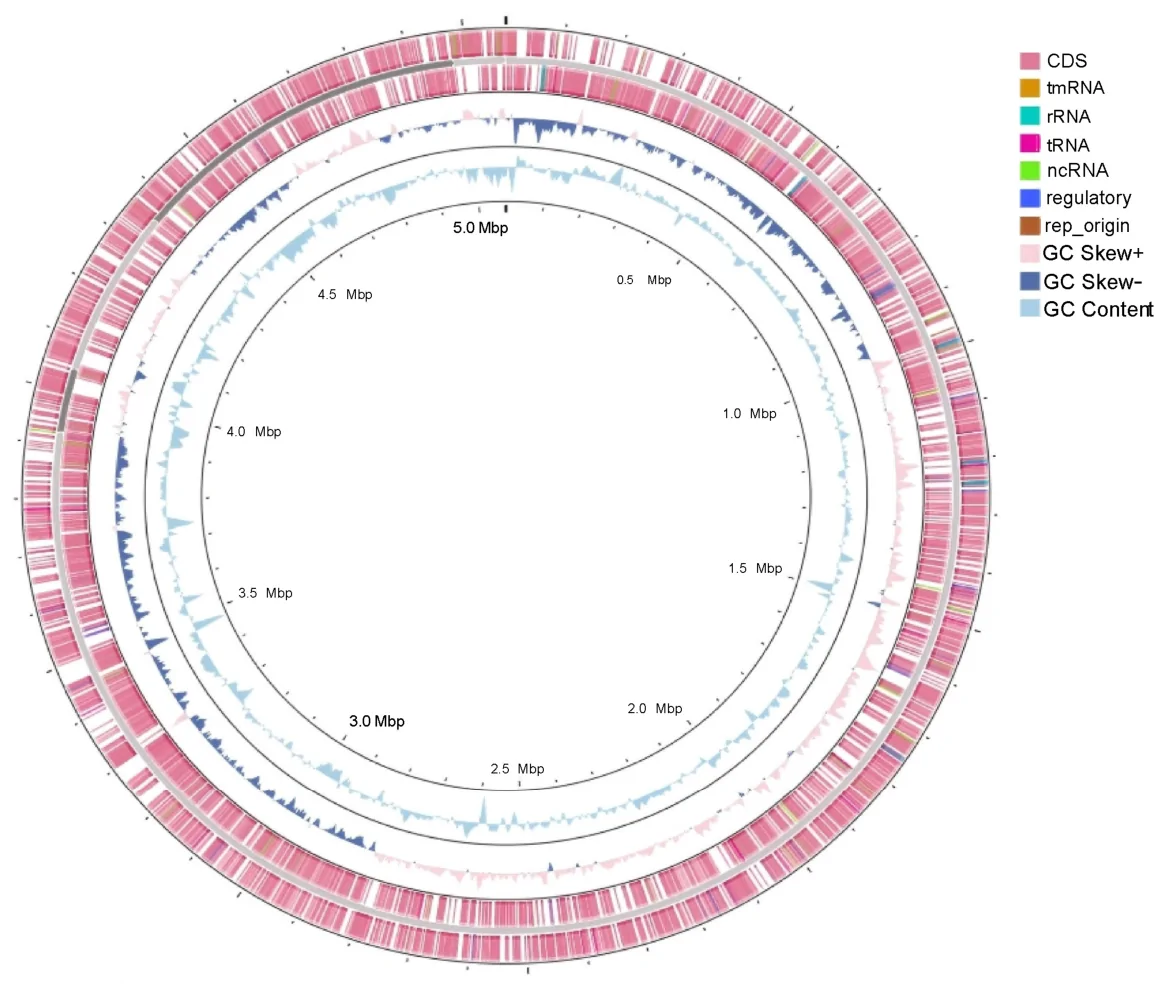
Circular genome map of *P. piersonii SZUGH1*. Circular representation of the complete genome of *P. piersonii SZUGH1* (one circular chromosome of 3,924,720 bp and four plasmid contigs). From the outermost to the innermost circles are coding sequences (CDSs) on the forward strand, coding sequences on the reverse strand (with tmRNA, rRNA, tRNA, ncRNA, regulatory features, and replication origins annotated), GC skew, and GC content. The GC content track indicates regions with relatively high or low GC content compared with the genome average, while the GC skew track represents the distribution of guanine and cytosine nucleotides across the chromosome. Genome coordinates are shown in megabase pairs (Mbp).

Antimicrobial susceptibility testing revealed that *P. piersonii* SZUGH1 exhibited broad antimicrobial susceptibility, including susceptibility to β-lactams, β-lactam/β-lactamase inhibitor combinations, aminoglycosides, tetracyclines, fluoroquinolones, and sulfonamides (Table 1). To further validate the data from MALDI-TOF MS and reveal the genomic characteristics of this strain, whole-genome sequencing was performed using a combination of PacBio long-read and Illumina short-read sequencing. The hybrid assembly yielded a complete genome of 5,016,492 bp with a GC content of 57.00%, comprising one circular chromosome (3,924,720 bp) and four plasmid contigs (pSZUGH1-1, 102,123 bp; pSZUGH1-2, 311,573 bp; pSZUGH1-3, 603,649 bp; pSZUGH1-4, 74,427 bp), with no gaps and an N50 of 3,924,720 bp (Figure 3, Table 2). Assembly quality was confirmed by CheckM2 (completeness 100%, contamination 1.26%). Genome annotation identified 4638 coding sequences, 80 tRNAs, 22 rRNAs, 2 tmRNAs, 46 ncRNAs, and one chromosomal origin of replication (*oriC*), with no CRISPR arrays (Table 2). Circularity of the chromosome and of plasmids pSZUGH1-1, pSZUGH1-3, and pSZUGH1-4 was supported by sequencing reads spanning the sequence-end junctions; pSZUGH1-2 is presented as a gap-free contig whose circularity could not be confirmed with either Illumina short-read or PacBio HiFi long-read data.

**Figure 4:**
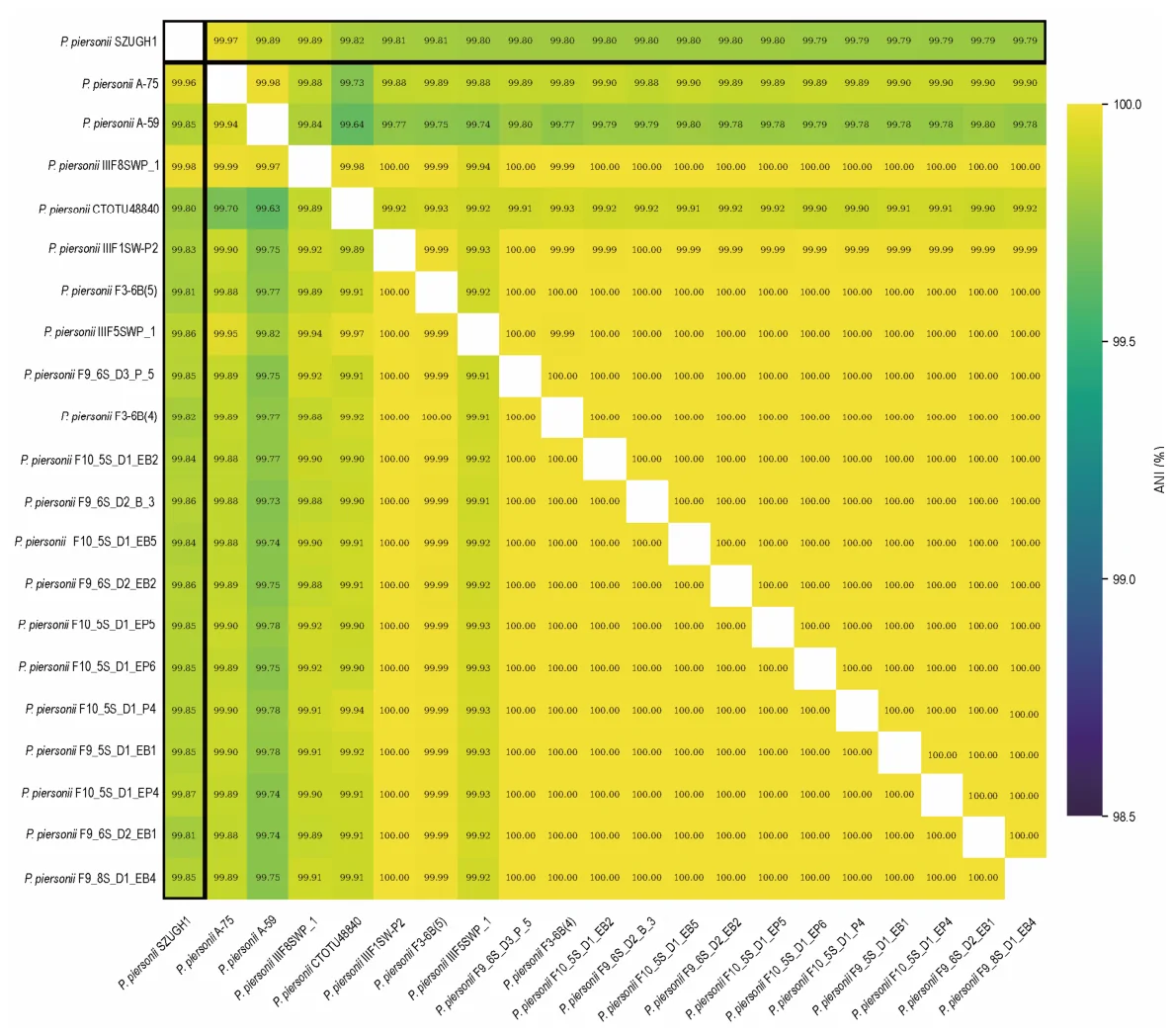
Pairwise average nucleotide identity (ANI) among *P. piersonii SZUGH1* and the 20 most closely related *Pantoea* genomes. Heatmap of pairwise ANI values calculated with fastANI; the color scale ranges from 98.5% to 100%, pairwise values are annotated in each cell, and self-comparisons on the diagonal are masked. SZUGH1 is indicated by a black frame.

ANI is a standard genomic metric for prokaryotic species delineation and is widely accepted for species-level assignment (>95–96%) [8]. Higher ANI values indicate a greater level of genome-wide sequence conservation and closer evolutionary relatedness among strains [9]. To determine the taxonomic position of strain *P. piersonii* SZUGH1, pairwise ANI values between SZUGH1 and 127 publicly available *Pantoea* and related genomes (Supplementary Table S1) were calculated using fastANI. The 20 genomes showing the highest ANI values are listed in Table 3. The full pairwise ANI matrix is provided in Supplementary Table S2. As shown in Figure 4, the closest relatives of SZUGH1 were *P. piersonii A-75* (ANI 99.97%) and *P. piersonii A-59* (ANI 99.89%), both well above the accepted species threshold of 95%. An ANI heatmap constructed from the top 20 genomes plus SZUGH1 showed that SZUGH1 clustered within the *P. piersonii* group. These results support the assignment of strain SZUGH1 to the species *P. piersonii* and are highly consistent with the identification obtained by MALDI-TOF MS.

**Figure 5:**
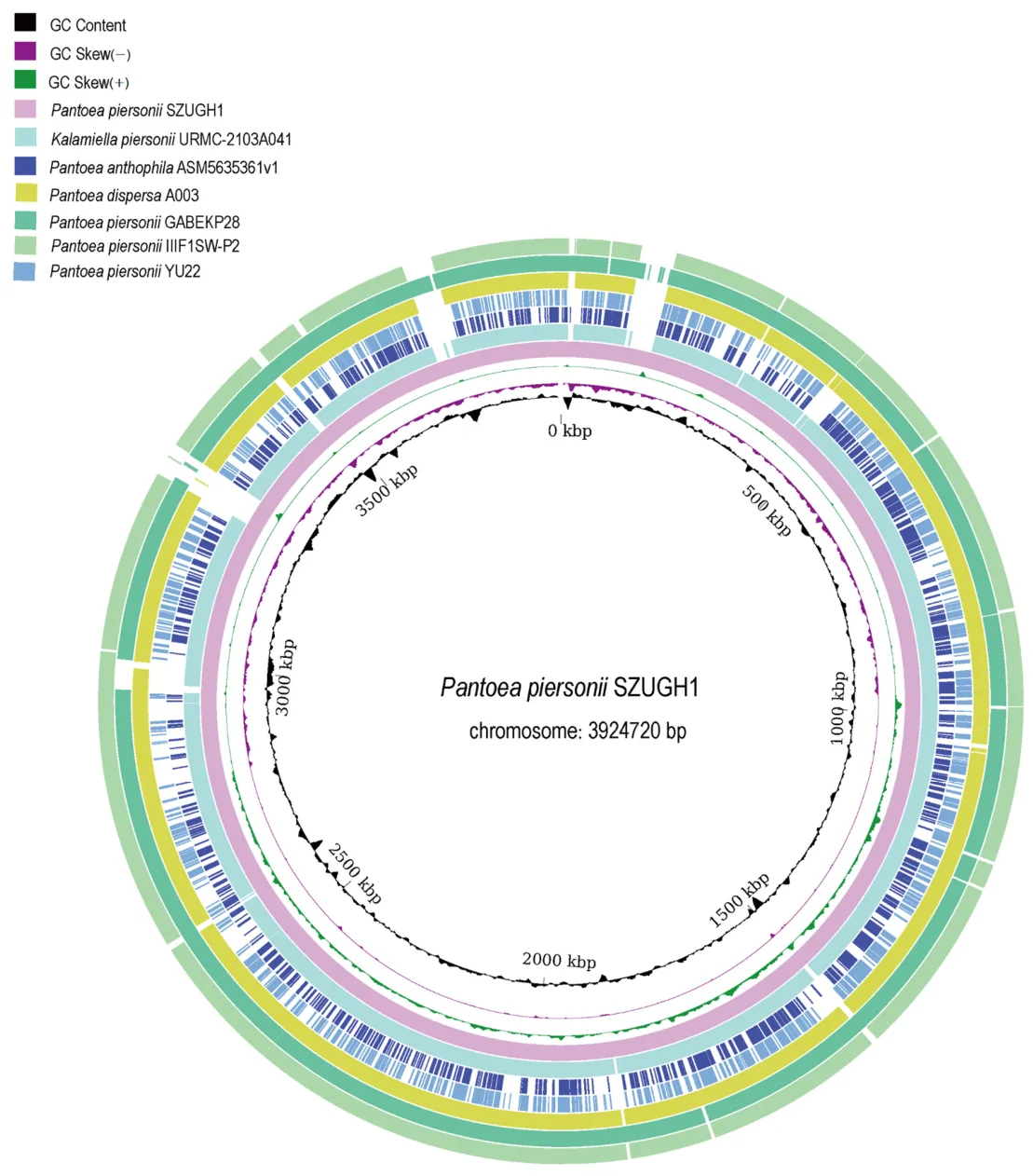
Whole-genome synteny analysis of *P. piersonii* SZUGH1 and related strains. Circular map of the *P. piersonii SZUGH1* chromosome (3,924,720 bp; central coordinate axis). From outside to inside: synteny blocks of *P. piersonii YU22*, IIIF1SW-P2, GABEKP28, *P. dispersa A003*, *P. anthophila ASM5635361v1*, and *P. piersonii URMC-2103A041* aligned against the SZUGH1 chromosome (one colored track per strain; alignments computed with NUCmer v4.0, delta-filter -1 -l 1000); the SZUGH1 reference ring (pink); GC skew (+) (green); GC skew (−) (purple); GC content (black, deviation from the mean); and the coordinate scale (kbp). Gaps in the comparator tracks indicate regions absent from or highly divergent in the respective strain.

To compare the genome organization of *P. piersonii* SZUGH1 with the selected reference strains, a whole-genome synteny analysis was performed using the *P. piersonii* SZUGH1 chromosome (3,924,720 bp) as the reference. The results showed a high degree of collinearity among the analyzed genomes (Figure 5). The *P. piersonii* strains GABEKP28, IIIF1SW-P2, and URMC-2103A041, together with *Pantoea* sp. YU22, exhibited the highest synteny with *P. piersonii SZUGH1*, with most genomic regions being highly conserved. In contrast, *P. anthophila ASM5635361v1* and *P. dispersa A003* displayed several discontinuous regions, indicating relatively greater genomic divergence. Overall, the synteny analysis revealed substantial conservation of genome structure among *P. piersonii* strains.

**Figure 6:**
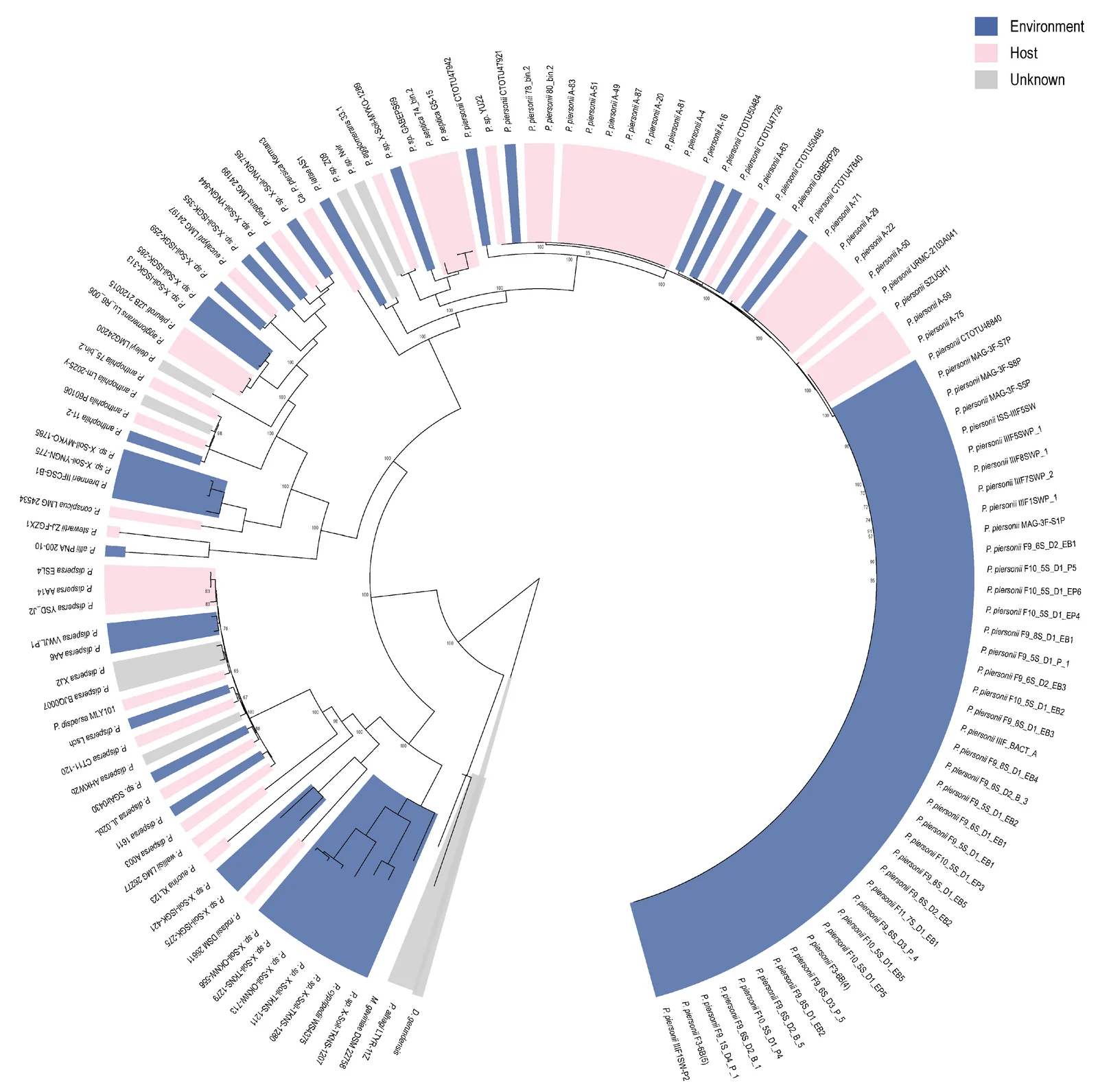
Core-genome phylogenetic tree of *P. piersonii SZUGH1* and related *Pantoea* strains. Maximum-likelihood tree inferred with IQ-TREE from the concatenated alignment of 1051 core genes across 128 genomes, with 1000 ultrafast bootstrap replicates. Duffyella gerundensis EM595 was used as the outgroup. Background shading indicates the isolation source of each clade: environmental (blue), host-associated (human/clinical, animal, or plant; pink), and unknown (gray). Bootstrap values ≥ 50 are shown at nodes.

To further determine the phylogenetic position of *P. piersonii SZUGH1*, a maximum-likelihood phylogenetic tree was reconstructed from a concatenated core-genome alignment of 1051 single-copy core genes (present in ≥99% of the 128 analyzed genomes and passing alignment quality filtering) inferred with Panaroo, using IQ-TREE with 1000 ultrafast bootstrap replicates and Duffyella gerundensis EM595 as the outgroup. Isolates were categorized according to their isolation source (human/clinical, environmental, plant, animal, or unknown) retrieved from BioSample metadata; for display in Figure 6, human/clinical, animal, and plant isolates are grouped as host-associated (pink), with environmental isolates in blue and isolates of unknown source in gray. As shown in Figure 6, *P. piersonii SZUGH1* clustered within the *P. piersonii* clade and showed the closest phylogenetic relationship with *P. piersonii A-75* and A-59, consistent with the ANI results. Within the broader phylogeny, *P. piersonii* strains formed a distinct monophyletic group separated from other *Pantoea* species, including *P. dispersa*, *P. anthophila*, and *P. agglomerans*. Notably, isolates from different ecological sources were interspersed within the *P. piersonii* clade, indicating that phylogenetic relatedness was not strictly associated with isolation source.

**Figure 7:**
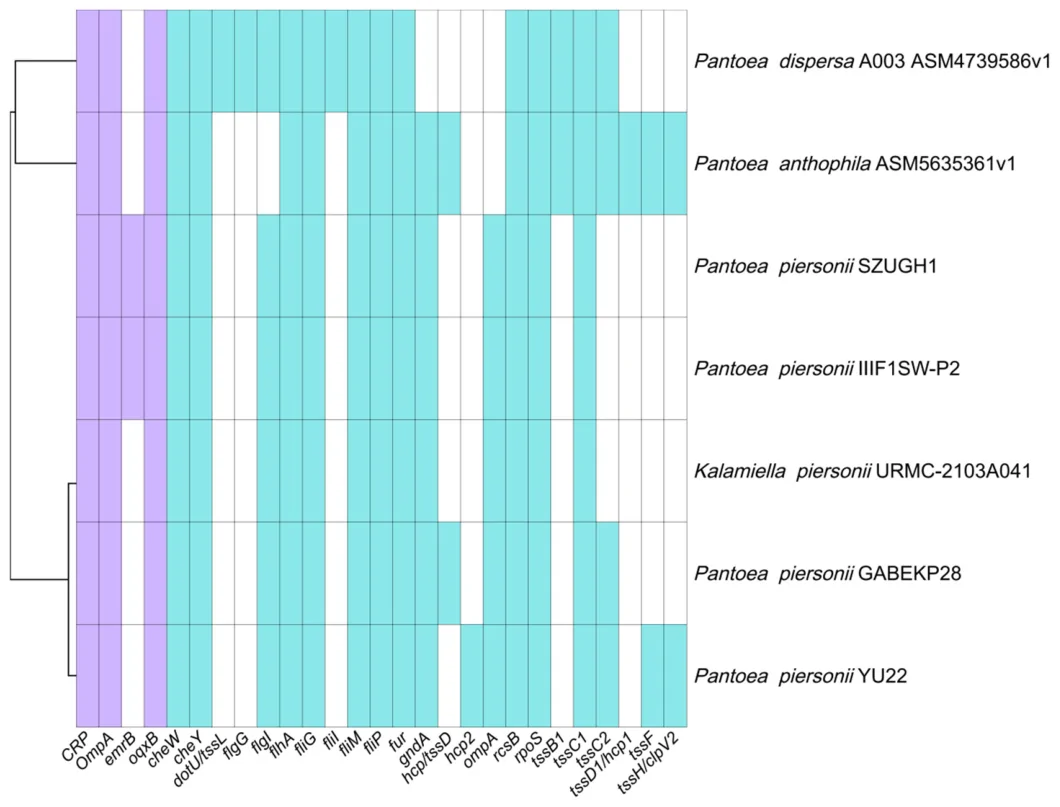
Distribution of predicted virulence factors and antimicrobial resistance genes among *Pantoea* strains. Rows represent bacterial strains and columns represent individual genes. Blue indicates putative virulence-associated genes predicted using the Virulence Factor Database (VFDB), whereas purple indicates putative antimicrobial resistance genes predicted using resistance gene databases. White indicates the absence of the corresponding genes.

We further predicted the virulence factors and antimicrobial resistance genes among closely related *Pantoea* strains (Figure 7). Hierarchical clustering based on gene presence/absence profiles demonstrated that *P. piersonii SZUGH1* clustered closely with *P. piersonii IIIF1SW-P2*, indicating a highly similar distribution pattern of both virulence-associated and antimicrobial resistance genes. Identified resistance-associated determinants in SZUGH1 included the global transcriptional regulator *CRP*, the outer membrane protein gene *OmpA*, and efflux pump genes *emrB* and *oqxB*. *OmpA* is associated with reduced outer membrane permeability, whereas emrB encodes a component of the EmrAB-TolC efflux system implicated in resistance to chloramphenicol and tetracycline [10]. In addition, *oqxB*, encoding a key subunit of the OqxAB-TolC efflux pump, has been linked to decreased susceptibility to fluoroquinolones and chloramphenicol [11]. Although chloramphenicol was not included in the antimicrobial susceptibility testing panel, tetracycline-class agents and fluoroquinolone activity were assessed, both of which showed full susceptibility in vitro (Table 1), suggesting that these efflux-associated determinants may be weakly expressed or not functionally dominant under clinical conditions. This highlights a potential discordance between genotypic resistance determinants and phenotypic expression, which has also been observed in other Enterobacterales species [12,13]. Overall, the phenotypic susceptibility profile was largely consistent with the predicted resistome, supporting a concordance between genomic resistance determinants and observed antimicrobial susceptibility in SZUGH1.

In addition, several virulence-associated genes were broadly conserved across the analyzed strains, including *cheW*, *fliG*, and *tssC1*. In contrast, certain genes exhibited strain-specific distribution (Figure 7). Notably, hcp2 was detected in 40 of the 128 analyzed genomes, including 16 *P. piersonii* strains, indicating intra-species diversification in secretion system components. Similar heterogeneity in virulence gene distribution has been reported in other *Pantoea* species, reflecting adaptation to diverse ecological niches [2]. Collectively, hierarchical clustering grouped SZUGH1 with other *P. piersonii* strains, suggesting a high degree of similarity in both virulence and antimicrobial resistance gene composition within this species.

## 5. Discussion

The genus *Pantoea* belongs to the order Enterobacterales and comprises Gram-negative bacilli widely distributed in environmental reservoirs, including plants, water, soil, and occasionally clinical settings [1]. Although members of the genus *Pantoea* are predominantly environmental organisms, increasing evidence indicates that they can act as opportunistic pathogens in immunocompromised patients, particularly those with indwelling medical devices such as central venous catheters or implantable ports [1,2,4]. As of June 2025, only a small number of human-associated infections have been reported, involving the urinary tract, bloodstream, gastrointestinal tract, and cardiac structures, highlighting its extreme rarity in clinical practice [14]. This case highlights the emerging opportunistic pathogenicity of *P. piersonii* and emphasizes the importance of integrating phenotypic and genomic approaches in rare bacterial infections.

To date, human infections caused by *P. piersonii* remain uncommon, with only a limited number of cases reported. Previous clinical manifestations have included urinary tract colonization/infection, bloodstream infection, gastrointestinal-associated bacteremia, cardiac infection, and soft tissue infection [5,6,15]. The first human-associated isolates were obtained from urine samples of a patient with kidney stone disease and from a patient with bacteremia, suggesting that this organism may colonize different human niches and cause invasive infection under favorable conditions [15,16]. Subsequent reports described bacteremia in a patient with underlying cardiovascular disease, diabetes mellitus, chronic obstructive pulmonary disease, and an indwelling catheter, indicating that impaired host conditions and invasive devices may represent important risk factors for infection [15]. In addition, a case of bacteremia in an infant with gastrointestinal food allergy demonstrated that *P. piersonii* can also cause invasive disease in patients without classical immunosuppression [6]. Collectively, these reports suggest that *P. piersonii* is an emerging opportunistic pathogen with a broad clinical spectrum, particularly affecting individuals with compromised host defenses, disrupted mucosal barriers, or exposure to invasive medical devices.

Phenotypically, *P. piersonii* exhibits biochemical characteristics closely resembling those of related species such as *Pantoea* septica, making conventional biochemical identification unreliable [1]. Moreover, 16S rRNA gene sequencing lacks sufficient discriminatory power within closely related members of the *Erwiniaceae*, further complicating accurate species-level identification [17,18]. Therefore, 16S rRNA gene sequencing was not performed in this study because it provides insufficient resolution for species-level identification among closely related members of the *Erwiniaceae* family. Closely related taxa within this group may share highly similar 16S rRNA gene sequences, limiting the ability of this approach to accurately distinguish *P. piersonii* from related species. In contrast, MALDI-TOF MS has recently improved clinical identification capability following inclusion of reference spectra for this species [19]. In the present case, MALDI-TOF MS yielded a high-confidence score (2.36/3.0), supporting reliable species-level identification. To further strengthen taxonomic resolution, whole-genome sequencing data from this isolate (third-generation sequencing) were integrated with publicly available *Pantoea* genomes for comparative genomic analysis. The ANI was consistent with species-level assignment to *P. piersonii*. Core-genome phylogenetic analysis further demonstrated that the isolate clustered tightly within the *P. piersonii* clade, clearly separated from other *Pantoea* and related *Erwiniaceae* taxa. These genomic findings provide robust confirmation of MALDI-TOF-based identification and highlight the importance of genome-informed taxonomy in rare environmental pathogens.

The antimicrobial susceptibility profile of SZUGH1 demonstrated broad susceptibility to most tested agents, including β-lactams, carbapenems, aminoglycosides, tetracyclines, fluoroquinolones, and polymyxins (Table 1). The phenotypic susceptibility profile was largely concordant with genomic findings, supporting a low resistance burden in SZUGH1. This is also clinically relevant, as it suggests that standard empiric therapies targeting Gram-negative bacteria remain effective against this organism in most cases. In addition, this phenotype is consistent with previous reports describing generally low resistance rates among *Pantoea* species, although occasional multidrug-resistant isolates have been reported in nosocomial settings [4]. Notably, cefoxitin showed intermediate susceptibility, which may reflect intrinsic variability in cephamycin activity among Enterobacterales [20].

Clinically, this case represents an extremely rare implantable venous access port-associated soft tissue infection caused by *P. piersonii* [5]. The patient had multiple predisposing factors for opportunistic infection, including advanced malignancy, a history of intensive systemic anticancer treatment with previous grade IV chemotherapy-induced myelosuppression, poor nutritional status, and long-term indwelling vascular access. Although specific cellular immune parameters, such as CD4-positive T-lymphocyte counts or lymphocyte subset analyses, were not assessed during this hospitalization, these clinical factors may have contributed to increased susceptibility to opportunistic infection [19]. Under conditions of impaired host immunity, organisms that are typically non-pathogenic may acquire the ability to cause clinically significant infections, particularly in the presence of foreign bodies such as intravascular devices [21,22]. Importantly, the infection in this case was identified intraoperatively in the absence of systemic inflammatory manifestations, underscoring the subtle clinical presentation of device-related infections caused by rare pathogens [5]. In the present case, complete removal of the infected implantable venous access port was the key source-control intervention and likely played the principal role in clinical resolution. Empirical antimicrobial therapy was administered as adjunctive treatment; however, the favorable outcome of a single case should not be interpreted as evidence of the efficacy of any specific antimicrobial agent [21]. This case therefore underscores the importance of prompt and complete device removal in the management of implantable port-associated infection.

In summary, this case represents, to the best of our knowledge, the first reported implantable venous access port-related infection caused by *P. piersonii*. It expands the known clinical spectrum of this emerging organism and emphasizes the necessity of integrating phenotypic microbiology with genome-based taxonomic validation. Early recognition, accurate microbial identification, and timely surgical intervention are essential for optimal management of such rare opportunistic infections.

## Figures and Tables

**Table 1:** Antimicrobial susceptibility testing of *P. piersonii* SZUGH1.

Antimicrobial Agent	Result	MIC Value (μg/mL)
Ceftazidime	Susceptible	≤1
Aztreonam	Susceptible	≤2
Ceftriaxone	Susceptible	≤1
Cefepime	Susceptible	≤1
Amoxicillin/Clavulanic acid	Susceptible	≤8/4
Piperacillin/Tazobactam	Susceptible	≤4/4
Cefoperazone/Sulbactam	Susceptible	≤0.5/8
Ampicillin/Sulbactam	Susceptible	8/4
Meropenem	Susceptible	0.25
Ertapenem	Susceptible	≤0.25
Gentamicin	Susceptible	≤2
Tobramycin	Susceptible	≤2
Trimethoprim/Sulfamethoxazole	Susceptible	≤1/19
Tetracycline	Susceptible	≤2
Tigecycline	Susceptible	≤1
Minocycline	Susceptible	2
Moxifloxacin	Susceptible	≤0.5
Colistin	Susceptible	≤1
Cefuroxime	Susceptible	8
Cefazolin	Susceptible	≤2
Cefoxitin	Intermediate	16

**Table 2:** Genomic features of *P. piersonii SZUGH1*.

Genomic Contents	Genome
Genome size (bp)	5,016,492
Number of contigs	5 (1 chromosome + 4 plasmids)
GC content (%)	57.00
N50 (bp)	3,924,720
N90 (bp)	311,573
N ratio (%)	0.00
Coding density (%)	86.25
Number of CDSs	4638
Number of pseudogenes	0
Number of hypothetical proteins	498
Number of tRNAs	80
Number of tmRNAs	2
Number of rRNAs	22
Number of ncRNAs	46
Number of ncRNA regions	43
Number of CRISPR arrays	0
Number of sORFs	1
Number of gaps	0
Number of oriCs	1
Number of oriVs	0
Number of oriTs	0

Abbreviation: CDS, coding sequence; sORF, small open reading frame; oriC, chromosomal origin of replication; oriV, plasmid origin of replication; oriT, origin of transfer.

**Table 3:** Genomic characteristics and ANI values of *P. piersonii SZUGH1* and the top 20 reference genomes.

No.	Species	Strain	Assembly Accession	BioSample	Genome Size (bp)	G + C (%)	ANI with *P. piersonii* SZUGH1 (%)
1	*Pantoea piersonii*	SZUGH1	-	SAMN61302005	5,016,492	57.00	100
2	*Pantoea piersonii*	A-75	GCF_055704995.1	SAMN56255786	5,004,457	57.0	99.97
3	*Pantoea piersonii*	A-59	GCF_055704505.1	SAMN56255775	5,581,698	56.5	99.89
4	*Pantoea piersonii*	IIIF8SWP_1	GCF_036880465.1	SAMN25944366	4,297,505	58.0	99.89
5	*Pantoea piersonii*	CTOTU48840	GCF_032061285.1	SAMN29161459	4,965,835	57.0	99.82
6	*Pantoea piersonii*	IIIF1SW-P2	GCF_003612015.1	SAMN10096957	4,851,875	57.0	99.82
7	*Pantoea piersonii*	F3-6B(5)	GCF_013403175.1	SAMN15344686	4,848,963	57.0	99.81
8	*Pantoea piersonii*	IIIF5SWP_1	GCF_036880505.1	SAMN25944363	4,548,605	57.5	99.80
9	*Pantoea piersonii*	F9_6S_D3_P_5	GCF_053623375.1	SAMN51431293	4,846,486	57.0	99.80
10	*Pantoea piersonii*	F3-6B(4)	GCF_013403225.1	SAMN15344685	4,847,371	57.0	99.80
11	*Pantoea piersonii*	F10_5S_D1_EB2	GCF_056280485.1	SAMN55954556	4,844,024	57.0	99.80
12	*Pantoea piersonii*	F9_6S_D2_B_3	GCF_053626035.1	SAMN51487955	4,847,019	57.0	99.80
13	*Pantoea piersonii*	F10_5S_D1_EB5	GCF_053625595.1	SAMN51482831	4,847,677	57.0	99.80
14	*Pantoea piersonii*	F9_6S_D2_EB2	GCF_053624415.1	SAMN51431526	4,847,089	57.0	99.80
15	*Pantoea piersonii*	F10_5S_D1_EP5	GCF_053625915.1	SAMN51483315	4,847,492	57.0	99.80
16	*Pantoea piersonii*	F10_5S_D1_EP6	GCF_053625515.1	SAMN51482859	4,847,384	57.0	99.79
17	*Pantoea piersonii*	F10_5S_D1_P4	GCF_053625015.1	SAMN51493196	4,847,130	57.0	99.79
18	*Pantoea piersonii*	F9_5S_D1_EB1	GCF_053623355.1	SAMN51431577	4,847,299	57.0	99.79
19	*Pantoea piersonii*	F10_5S_D1_EP4	GCF_053625535.1	SAMN51432686	4,847,277	57.0	99.79
20	*Pantoea piersonii*	F9_6S_D2_EB1	GCF_053626475.1	SAMN51432529	4,847,211	57.0	99.79
21	*Pantoea piersonii*	F9_8S_D1_EB4	GCF_053624575.1	SAMN51432682	4,847,100	57.0	99.79

## Data Availability

The sequences of the isolates are available from the Sequence Read Archive (SRA) maintained by the National Center for Biotechnology Information (NCBI) under project number: PRJNA1489473. The Illumina short-read data are deposited under SRA accession SRR39447605, and the PacBio long-read data are being deposited under the same BioProject.

## Supplementary Materials

The following supporting information can be downloaded at https://www.mdpi.com/article/10.3390/microorganisms14091996/s1. Table S1: Reference genomes used in the comparative genomic and phylogenetic analyses. Table S2: Full pairwise average nucleotide identity (ANI) matrix of *P. piersonii* SZUGH1 and the top 20 reference genomes (values in %).
