# Supplementary material for: Catheter-Associated Soft Tissue Infection Caused by Pantoea piersonii in a Totally Implantable Venous Access Port: The First Report from China
Source: Microorganisms. 2026 Sep 9;14(9):1996. doi: 10.3390/microorganisms14091996 (PMC13609269; doi:10.3390/microorganisms14091996)
Supplement: Supplementary file 1 [file microorganisms-14-01996-s001.zip › microorganisms-4468015-supplementary.pdf]

Table S1. Reference genomes used in the comparative genomic and phylogenetic analyses.

No.	Organism	Strain	Assembly accession	Isolation source	ANI with SZUGH1 (%)
1	*Pantoea piersonii*	SZUGH1 [a]	-	Host-associated (human/clinical)	100.0000
2	*Pantoea piersonii*	A-75	GCF_055704995.1	Host-associated (human/clinical)	99.9664
3	*Pantoea piersonii*	A-59	GCF_055704505.1	Host-associated (human/clinical)	99.8911
4	*Pantoea piersonii*	IIIF8SWP_1	GCF_036880465.1	Environmental	99.8891
5	*Pantoea piersonii*	CTOTU48840	GCF_032061285.1	Environmental	99.8228
6	*Pantoea piersonii*	IIIF1SW-P2	GCF_003612015.1	Environmental	99.8150
7	*Pantoea piersonii*	F3-6B(5)	GCF_013403175.1	Environmental	99.8116
8	*Pantoea piersonii*	IIIF5SWP_1	GCF_036880505.1	Environmental	99.8039
9	*Pantoea piersonii*	F9_6S_D3_P_5	GCF_053623375.1	Environmental	99.8037
10	*Pantoea piersonii*	F3-6B(4)	GCF_013403225.1	Environmental	99.8026
11	*Pantoea piersonii*	F10_5S_D1_EB2	GCF_056280485.1	Environmental	99.7998
12	*Pantoea piersonii*	F9_6S_D2_B_3	GCF_053626035.1	Environmental	99.7995
13	*Pantoea piersonii*	F10_5S_D1_EB5	GCF_053625595.1	Environmental	99.7980
14	*Pantoea piersonii*	F9_6S_D2_EB2	GCF_053624415.1	Environmental	99.7959
15	*Pantoea piersonii*	F10_5S_D1_EP5	GCF_053625915.1	Environmental	99.7952
16	*Pantoea piersonii*	F10_5S_D1_EP6	GCF_053625515.1	Environmental	99.7936
17	*Pantoea piersonii*	F10_5S_D1_P4	GCF_053625015.1	Environmental	99.7932
18	*Pantoea piersonii*	F9_5S_D1_EB1	GCF_053623355.1	Environmental	99.7920
19	*Pantoea piersonii*	F10_5S_D1_EP4	GCF_053625535.1	Environmental	99.7872
20	*Pantoea piersonii*	F9_6S_D2_EB1	GCF_053626475.1	Environmental	99.7864
21	*Pantoea piersonii*	F9_8S_D1_EB4	GCF_053624575.1	Environmental	99.7861
22	*Pantoea piersonii*	F9_1S_D4_P_1	GCF_053624795.1	Environmental	99.7861
23	*Pantoea piersonii*	F9_6S_D2_B_5	GCF_053625375.1	Environmental	99.7858
24	*Pantoea piersonii*	F11_7S_D1_EB1	GCF_053626335.1	Environmental	99.7854
25	*Pantoea piersonii*	F9_6S_D2_EB3	GCF_053625355.1	Environmental	99.7850
26	*Pantoea piersonii*	F10_5S_D1_EP3	GCF_053624455.1	Environmental	99.7845
27	*Pantoea piersonii*	F9_8S_D1_EB1	GCF_053625195.1	Environmental	99.7845
28	*Pantoea piersonii*	F9_6S_D1_EB1	GCF_053624835.1	Environmental	99.7831
29	*Pantoea piersonii*	F9_5S_D1_P_1	GCF_053625615.1	Environmental	99.7828
30	*Pantoea piersonii*	F9_8S_D1_EB2	GCF_053624555.1	Environmental	99.7812
31	*Pantoea piersonii*	IIIF7SWP_2	GCF_036880485.1	Environmental	99.7796
32	*Pantoea piersonii*	F9_6S_D3_P_4	GCF_053624115.1	Environmental	99.7771
33	*Pantoea piersonii*	F9_8S_D1_EB3	GCF_053626455.1	Environmental	99.7768
34	*Pantoea piersonii*	F9_5S_D1_EB2	GCF_053626875.1	Environmental	99.7766
35	*Pantoea piersonii*	IIIF_BACT_A	GCF_013403215.1	Environmental	99.7760
36	*Pantoea piersonii*	F9_6S_D2_B_1	GCF_053623095.1	Environmental	99.7719
37	*Pantoea piersonii*	F10_5S_D1_P5	GCF_053623515.1	Environmental	99.7644
38	*Pantoea piersonii*	F9_8S_D1_EB5	GCF_053624075.1	Environmental	99.7608
39	*Pantoea piersonii*	MAG-3F-S5P	GCF_020092965.1	Environmental	99.7019
40	*Pantoea piersonii*	MAG-3F-S7P	GCF_020092915.1	Environmental	99.7009
41	*Pantoea piersonii*	missing	GCF_003327525.1	Environmental	99.6990
42	*Pantoea piersonii*	MAG-3F-S1P	GCA_020093055.1	Environmental	99.6932
43	*Pantoea piersonii*	MAG-3F-S8P	GCF_020092925.1	Environmental	99.6725
44	*Pantoea piersonii*	IIIF1SWP_1	GCF_036880625.1	Environmental	99.6692
45	*Pantoea piersonii*	A-49	GCF_055704725.1	Host-associated (human/clinical)	99.0530
46	*Pantoea piersonii*	GABEKP28	GCF_027570015.1	Host-associated (human/clinical)	98.9327
47	*Pantoea sp. YU22*	YU22	GCF_003970755.1	Host-associated (human/clinical)	98.9180
48	*Pantoea piersonii*	CTOTU50484	GCF_032101565.1	Environmental	98.9169

49	*Pantoea piersonii*	URMC-2103A041	GCF_027920325.1	Host-associated (human/clinical)	98.8752
50	*Pantoea piersonii*	CTOTU50495	GCF_032101165.1	Environmental	98.8448
51	*Pantoea piersonii*	80_bin.2	GCF_056353895.1	Host-associated (human/clinical)	98.8427
52	*Pantoea piersonii*	78_bin.2	GCF_056352775.1	Host-associated (human/clinical)	98.8365
53	*Pantoea piersonii*	A-16	GCF_055705305.1	Host-associated (human/clinical)	98.8007
54	*Pantoea piersonii*	A-81	GCF_055704885.1	Host-associated (human/clinical)	98.8001
55	*Pantoea piersonii*	A-4	GCF_055705245.1	Host-associated (human/clinical)	98.7971
56	*Pantoea piersonii*	A-51	GCF_055704705.1	Host-associated (human/clinical)	98.7911
57	*Pantoea piersonii*	CTOTU47640	GCF_032016845.1	Environmental	98.7883
58	*Pantoea piersonii*	A-87	GCF_055704925.1	Host-associated (human/clinical)	98.7860
59	*Pantoea piersonii*	A-20	GCF_055705285.1	Host-associated (human/clinical)	98.7825
60	*Pantoea piersonii*	A-83	GCF_055704485.1	Host-associated (human/clinical)	98.7802
61	*Pantoea piersonii*	A-71	GCF_055705085.1	Host-associated (human/clinical)	98.7421
62	*Pantoea piersonii*	A-50	GCF_055704685.1	Host-associated (human/clinical)	98.7350
63	*Pantoea piersonii*	A-29	GCF_055705165.1	Host-associated (human/clinical)	98.7226
64	*Pantoea piersonii*	A-22	GCF_055704805.1	Host-associated (human/clinical)	98.7151
65	*Pantoea piersonii*	CTOTU47921	GCF_031986405.1	Environmental	98.7116
66	*Pantoea piersonii*	A-63	GCF_055705045.1	Host-associated (human/clinical)	98.5131
67	*Pantoea piersonii*	CTOTU47726	GCF_032014945.1	Environmental	98.3799
68	*Pantoea piersonii*	CTOTU47942	GCF_036726665.1	Environmental	96.0282
69	*Pantoea sp. X-Soil-MYKO-1289*	X-Soil-MYKO-1289	GCF_059730625.1	Environmental	90.4273
70	*Pantoea sp. GABEPS69*	GABEPS69	GCF_045780835.1	Host-associated (human/clinical)	90.4221
71	*Pantoea sp. Z09*	Z09	GCF_022394795.1	Unknown	90.1455
72	*Pantoea septica*	G5-15	GCF_055809015.1	Host-associated (plant)	90.1146
73	*Pantoea septica*	74_bin.2	GCF_056353495.1	Host-associated (human/clinical)	90.0374
74	*Pantoea agglomerans*	33.1	GCF_020149765.1	Host-associated (plant)	89.9066
75	*Pantoea sp. Nvir*	Nvir	GCF_963853465.1	Unknown	89.8788
76	*Pantoea latae*	AS1	GCF_002077695.1	Environmental	89.4243
77	Candidatus *Pantoea persica*	Kerman3	GCF_013753875.1	Host-associated (animal)	86.3996
78	*Pantoea dispersa*	BJQ0007	GCF_009362975.1	Unknown	82.5525
79	*Pantoea dispersa*	MLY101	GCF_037039485.1	Host-associated (plant)	82.4886
80	*Pantoea dispersa*	ESL4	GCF_025919605.1	Host-associated (plant)	82.4566
81	*Pantoea dispersa*	JL.02bL	GCF_025853815.1	Host-associated (plant)	82.4559
82	*Pantoea sp. SGAir0430*	SGAir0430	GCF_043549295.1	Environmental	82.4497
83	*Pantoea dispersa*	CT11-120	GCF_047325125.1	Host-associated	82.4494

				(plant)	
84	*Pantoea dispersa*	YSD_J2	GCF_022220865.1	Host-associated (plant)	82.4443
85	*Pantoea dispersa*	AA14	GCF_054611875.1	Host-associated (plant)	82.4400
86	*Pantoea dispersa*	VWJL.P1	GCF_028993655.1	Environmental	82.4189
87	*Pantoea dispersa*	AA6	GCF_054611835.1	Environmental	82.4170
88	*Pantoea dispersa*	XJ2	GCF_040207715.1	Unknown	82.3918
89	*Pantoea dispersa*	AHKW2b	GCF_019890975.1	Unknown	82.3870
90	*Pantoea dispersa*	Lsch	GCF_019890955.1	Environmental	82.3769
91	*Pantoea dispersa*	A003	GCF_047395865.1	Host-associated (human/clinical)	82.3075
92	*Pantoea dispersa*	1611	GCF_059731165.1	Environmental	82.2700
93	*Pantoea deleyi*	LMG24200	GCF_022647325.1	Unknown	82.1624
94	*Pantoea sp. X-Soil-YNGN-775*	X-Soil-YNGN-775	GCF_059730575.1	Environmental	82.0541
95	*Pantoea anthophila*	P60106	GCF_060078235.1	Host-associated (human/clinical)	82.0393
96	*Pantoea sp. X-Soil-TKNS-1207*	X-Soil-TKNS-1207	GCF_059730485.1	Environmental	82.0023
97	*Pantoea brenneri*	IIFCSG-B1	GCF_013403315.1	Environmental	81.9797
98	*Pantoea sp. X-Soil-MYKO-1785*	X-Soil-MYKO-1785	GCF_059730675.1	Environmental	81.9692
99	*Pantoea anthophila*	11-2	GCF_000969395.1	Environmental	81.9015
100	*Pantoea conspicua*	LMG 24534	GCF_002095315.1	Host-associated (human/clinical)	81.9001
101	*Pantoea anthophila*	Lm-2025-y	GCF_059718695.1	Unknown	81.8947
102	*Pantoea vagans*	LMG 24199	GCF_004792415.1	Host-associated (plant)	81.8482
103	*Pantoea agglomerans*	Lu_R6_006	GCF_051136215.1	Host-associated (plant)	81.8134
104	*Pantoea anthophila*	75_bin.2	GCF_056353615.1	Host-associated (human/clinical)	81.7981
105	*Pantoea sp. X-Soil-ISGK-285*	X-Soil-ISGK-285	GCF_059730815.1	Environmental	81.7310
106	*Pantoea sp. X-Soil-ISGK-313*	X-Soil-ISGK-313	GCF_059730755.1	Environmental	81.6675
107	*Pantoea sp. X-Soil-YNGN-844*	X-Soil-YNGN-844	GCF_059730595.1	Environmental	81.6544
108	*Pantoea sp. X-Soil-YNGN-785*	X-Soil-YNGN-785	GCF_059730585.1	Environmental	81.6263
109	*Pantoea sp. X-Soil-ISGK-355*	X-Soil-ISGK-355	GCF_059730805.1	Environmental	81.5772
110	*Pantoea pleuroti*	JZB 2120015	GCF_014156615.1	Host-associated (plant)	81.5726
111	*Mixta gaviniae*	DSM 22758	GCF_002953195.1	Unknown	81.5473
112	*Pantoea wallisii*	LMG 26277	GCF_002095485.1	Host-associated (plant)	81.4880
113	*Pantoea eucrina*	XL123	GCF_020079945.1	Host-associated (plant)	81.4683
114	*Pantoea eucalypti*	LMG 24197	GCF_009646115.1	Host-associated (animal)	81.4004
115	*Pantoea sp. X-Soil-ISGK-275*	X-Soil-ISGK-275	GCF_059730795.1	Environmental	81.3836
116	*Pantoea sp. X-Soil-ISGK-259*	X-Soil-ISGK-259	GCF_059730705.1	Environmental	81.3128
117	*Pantoea cypripedii*	WS4375	GCF_017875755.1	Environmental	81.2659
118	*Pantoea sp. X-Soil-TKNS-1280*	X-Soil-TKNS-1280	GCF_059730475.1	Environmental	81.1795

119	*Pantoea sp. X-Soil-ISGK-421*	X-Soil-ISGK-421	GCF_059730785.1	Environmental	81.1013
120	*Pantoea sp. X-Soil-TKNS-1279*	X-Soil-TKNS-1279	GCF_059730565.1	Environmental	81.0901
121	*Pantoea sp. X-Soil-TKNS-1211*	X-Soil-TKNS-1211	GCF_059730495.1	Environmental	81.0825
122	*Pantoea sp. X-Soil-OKNW-556*	X-Soil-OKNW-556	GCF_059730695.1	Environmental	81.0584
123	*Pantoea sp. X-Soil-OKNW-713*	X-Soil-OKNW-713	GCF_059730685.1	Environmental	81.0458
124	*Pantoea rodasii*	DSM 26611	GCF_002811195.1	Host-associated (plant)	80.7026
125	*Pantoea stewartii*	ZJ-FGZX1	GCF_011044475.1	Host-associated (plant)	80.4108
126	*Pantoea alhagi*	LTYR-11Z	GCF_002101395.1	Unknown	80.2156
127	*Duffyella gerundensis*	EM595	GCF_001517405.1	Unknown	80.1766
128	*Pantoea allii*	PNA 200-10	GCF_003148935.1	Environmental	80.0782

[a] *Pantoea piersonii* SZUGH1 was isolated in this study; raw sequencing data are available under BioProject PRJNA1489473 (SRA accession SRR39447605). Reference genomes are sorted by descending ANI with SZUGH1.

Table S2. Full pairwise average nucleotide identity (ANI) matrix of *P. piersonii* SZUGH1 and the top 20 reference genomes (values in %).

	**P. piersonii SZUGH1**	**P. piersonii A-75**	**P. piersonii A-59**	**P. piersonii IIIF8SWP_1**	**P. piersonii CTOTU48840**	**P. piersonii IIIF1SW-P2**	**P. piersonii F3-6B (5)**	**P. piersonii IIIF5SWP_1**	**P. piersonii F9_6S_D3_P_5**	**P. piersonii F3-6B (4)**	**P. piersonii F10_5S_D1_EB2**	**P. piersonii F9_6S_D2_B_3**	**P. piersonii F10_5S_D1_EB5**	**P. piersonii F9_6S_D2_EB2**	**P. piersonii F10_5S_D1_EP5**	**P. piersonii F10_5S_D1_EP6**	**P. piersonii F10_5S_D1_P4**	**P. piersonii F9_5S_D1_EB1**	**P. piersonii F10_5S_D1_EP4**	**P. piersonii F9_6S_D2_EB1**	**P. piersonii F9_8S_D1_EB4**
**P. piersonii SZUGH1**	100.0	99.9664	99.8911	99.8891	99.8228	99.8115	99.8116	99.8039	99.8037	99.8026	99.7998	99.7995	99.798	99.7959	99.7952	99.7936	99.7932	99.792	99.7872	99.7864	99.7861
**P. piersonii A-75**	99.9579	100.0	99.9835	99.8799	99.7271	99.877	99.8919	99.8791	99.8922	99.8899	99.8971	99.8827	99.8972	99.8866	99.89	99.8949	99.8948	99.8951	99.8952	99.8991	99.8962
**P. piersonii A-59**	99.8535	99.9401	100.0	99.8378	99.6381	99.7692	99.7534	99.7434	99.8039	99.7696	99.7902	99.7917	99.8038	99.7843	99.7841	99.7905	99.7806	99.7822	99.7756	99.7975	99.7783
**P. piersonii IIIF8SWP_1**	99.9822	99.9874	99.9731	100.0	99.9798	99.9952	99.9908	99.9378	99.9958	99.9943	99.9955	99.9958	99.9955	99.9956	99.9955	99.9958	99.9955	99.9958	99.9958	99.9958	99.9955
**P. piersonii CTOT**	99.795	99.7016	99.6262	99.892	100.0	99.9173	99.9314	99.9155	99.9132	99.9278	99.9171	99.9222	99.9139	99.9224	99.9154	99.9011	99.9011	99.9142	99.9141	99.9036	99.9161

**U488840**																				
**P. piersonii IIIF1SW-P2**	99.8282	99.8987	99.7539	99.9227	99.8941	100.0	99.9898	99.9255	99.9956	99.9948	99.9893	99.995	99.9946	99.9908	99.9903	99.9942	99.9904	99.9902	99.9905	99.9937	99.9941
**P. piersonii F3-6B(5)**	99.8148	99.8828	99.7675	99.8877	99.9084	99.9991	100.0	99.9199	99.9996	99.9997	99.9991	99.9981	99.9996	99.9992	99.9996	99.9992	99.9992	99.9997	99.9996	99.9987	99.9995
**P. piersonii IIIF5SWP_1**	99.8604	99.955	99.8195	99.9353	99.9656	99.9958	99.9935	100.0	99.9967	99.9915	99.9959	99.9966	99.9964	99.9965	99.9959	99.9961	99.9959	99.9966	99.9966	99.9966	99.9961
**P. piersonii F9_6S_D3_P_5**	99.8541	99.8875	99.7475	99.9249	99.906	99.9986	99.9902	99.9085	100.0	99.9978	99.9998	99.9984	99.9998	99.9998	99.9998	99.9998	99.9997	99.9998	99.9999	99.9995	99.9997
**P. piersonii F3-6B(4)**	99.8223	99.888	99.7661	99.8837	99.9199	99.9987	99.9955	99.9108	99.9993	100.0	99.9997	99.9978	99.9993	99.9997	99.9992	99.9997	99.9997	99.9993	99.9992	99.9992	99.999
**P. piersonii F10_5S_D1_EB2**	99.842	99.8816	99.7657	99.9029	99.8993	99.998	99.9895	99.9183	99.9996	99.9964	100.0	99.9978	99.9996	99.9996	99.9996	99.9996	99.9996	99.9996	99.9997	99.9992	99.9995
**P. piersonii F9_6S_D2_B_3**	99.8581	99.8811	99.7312	99.8782	99.9011	99.9986	99.989	99.9113	99.9988	99.9961	99.9987	100.0	99.9997	99.9997	99.9996	99.9996	99.9996	99.9997	99.9997	99.9998	99.9997
**P. piersonii F10_5S_D1_EB5**	99.8383	99.877	99.7445	99.903	99.9104	99.9987	99.989	99.9189	99.9998	99.9971	99.9997	99.9983	100.0	99.9998	99.9998	99.9998	99.9993	99.9998	99.9999	99.9999	99.9999
**P. piersonii F9_6S_D2_EB2**	99.858	99.89	99.7477	99.8843	99.9105	99.9981	99.9876	99.9203	99.9987	99.9961	99.9987	99.9997	99.9996	100.0	99.9997	99.9995	99.9997	99.9997	99.9996	99.9997	99.9993
**P. piersonii F10_5S_D1_E**	99.8508	99.8988	99.7761	99.9162	99.9042	99.9986	99.9899	99.9307	99.9997	99.9974	99.9997	99.9982	99.9998	99.9999	100.0	99.9998	99.9994	99.9998	99.9999	99.9998	99.9997

**P5**																					
**P. piersonii F10_5S_D1_EP6**	99.8496	99.8906	99.7517	99.9173	99.8994	99.9985	99.9903	99.9284	99.9992	99.9975	99.9991	99.9982	99.9997	99.9997	99.9998	100.0	99.9997	99.9998	99.9998	99.9998	99.9994
**P. piersonii F10_5S_D1_P4**	99.8494	99.899	99.7793	99.9079	99.9368	99.9983	99.9892	99.9346	99.9988	99.9959	99.9989	99.9982	99.9998	99.9998	99.9998	99.9998	100.0	99.9998	99.9998	99.9998	99.9996
**P. piersonii F9_5S_D1_EB1**	99.8501	99.9001	99.7766	99.906	99.9209	99.9986	99.9891	99.928	99.9989	99.9961	99.9989	99.9983	99.9997	99.9998	99.9998	99.9998	99.9998	100.0	99.9998	99.9998	99.9997
**P. piersonii F10_5S_D1_EP4**	99.8692	99.8851	99.7442	99.9004	99.905	99.9986	99.9883	99.9299	99.9998	99.9965	99.9998	99.9983	99.9999	99.9998	99.9999	99.9999	99.9993	99.9999	100.0	99.9999	99.9999
**P. piersonii F9_6S_D2_EB1**	99.8142	99.8784	99.7449	99.8867	99.9051	99.9951	99.9889	99.9241	99.9997	99.997	99.9996	99.9998	99.9998	99.9997	99.9997	99.9998	99.9993	99.9998	99.9998	100.0	99.9997
**P. piersonii F9_8S_D1_EB4**	99.8531	99.8865	99.7452	99.9118	99.9062	99.9987	99.9905	99.9167	99.9998	99.9975	99.9997	99.9984	99.9999	99.9997	99.9998	99.9998	99.9992	99.9998	99.9999	99.9999	100.0

Pairwise ANI values were computed with fastANI. Rows correspond to query genomes and columns to reference genomes; because fastANI ANI estimates are query-dependent, the matrix is not perfectly symmetric (e.g., SZUGH1 vs. A-75 = 99.9664% and A-75 vs. SZUGH1 = 99.9579%). Self-comparisons (diagonal) are 100.0%.
